# Difficulty in Removing a Ureteral Stent post Childbirth due to Encrustation: A Case Report

**DOI:** 10.24546/0100490328

**Published:** 2024-07-01

**Authors:** MASATO YANAGI, HIROYOSHI KONO, HIROYUKI MURAMATSU, AKIFUMI KATSU, RYOJI KIMATA, TSUTOMU HAMASAKI, YUKIHIRO KONDO

**Affiliations:** 1 Department of Urology, Nippon Medical School Musashikosugi Hospital, Kanagawa, Japan; 2 Department of Radiology, Nippon Medical School Musashikosugi Hospital, Kanagawa, Japan; 3 Department of Urology, Nippon Medical School Hospital, Tokyo, Japan

**Keywords:** Encrustation, Lithotripsy, Pregnant, Ureteral stent, Transurethral lithotripsy, Case report

## Abstract

**BACKGROUND:**

We present a case involving a pregnant woman who needed transurethral lithotripsy for ureteral stent removal because of the stent encrustation.

**CLINICAL CASE:**

A 34-year-old woman was diagnosed with calculous pyelonephritis, and a double-loop ureteral stent was placed in her right ureter, after which the pyelonephritis resolved. One week after her delivery, we attempted to remove the ureteral stent; however, the encrustation of the proximal and distal coils made it impossible. We then crushed the encrustation by transurethral lithotripsy and removed the ureteral stent successfully. The encrustation component was calcium phosphate, and the urinary pH during pregnancy and after delivery was 7.5.

**CONCLUSION:**

Even in pregnant patients, patients placed ureteral stents for obstructive pyelonephritis with high urine pH might need to be replaced in the short term due to concerns regarding phosphate encrustation.

Ureteral stenting is a common treatment for relieving ureteral obstruction and is effective for treating symptomatic ureteral obstruction when conservative management fails (1). However, indwelling ureteral stents, especially those with prolonged indwelling times, can cause various complications including ureteral stent encrustation (2). The replacement of a ureteral stent in pregnant women might expose the fetus to radiation; therefore, psychological hurdles for performing ureteral stents replacement are high. Hence, ureteral stent replacement may not be performed until delivery, even if it is for a period of approximately 3–4 months.

Here, we report the case of ureteral stent encrustation necessitating transurethral lithotripsy (TUL) in a pregnant woman for ureteral stent removal, 13 weeks after its initial placement. Informed consent for publication has been obtained from the patient described in this case report.

## CLINICAL CASE

**Figure 1:**
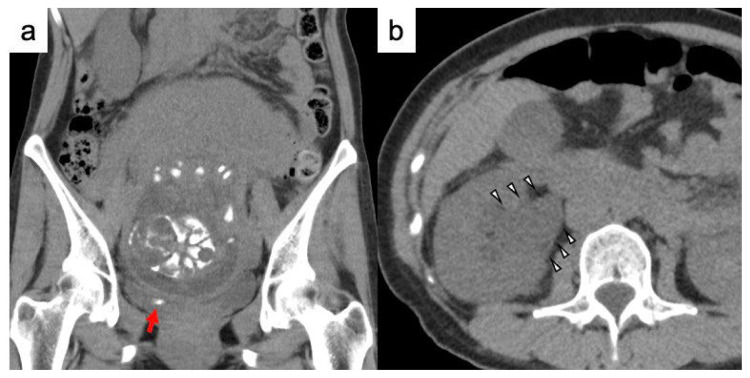
Computed tomography (CT) findings before placement of a ureteral stent a. The red arrow shows a ureteral stone. b. The white arrow heads show hydronephrosis.

A 34-year-old woman developed a fever of 39°C and lower back pain at 26 weeks and 6 days of pregnancy. Her white blood cell count was elevated at 17,000/μL, and her serum creatinine level was 0.61 mg/dL. Her urine sediment showed pyuria, and computed tomography (CT) revealed a 5 mm urinary stone at right ureterovesical junction. Thus, she was diagnosed with calculous pyelonephritis (Figure. 1a, 1b). CT also revealed multiple tiny urinary stones at left renal pelvis. Her serum calcium level and intact parathyroid hormone were normal at 9.8 mg/dL and 38 pg/ml. She has no medical history.

**Figure 2:**
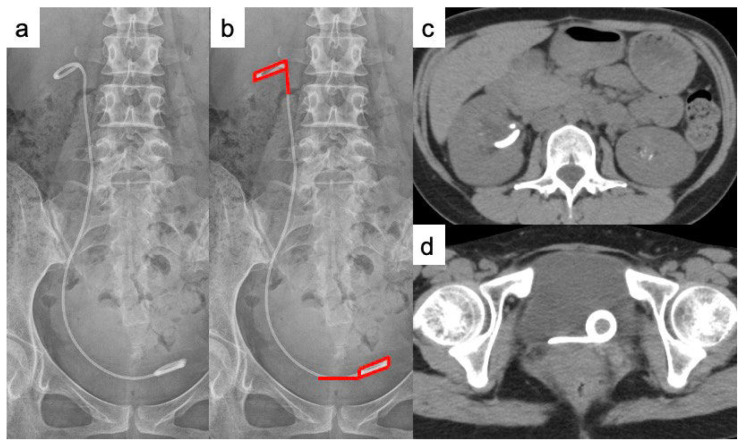
Findings of KUB and CT 13 weeks after placement of a ureteral stent a. Ureteral stent encrustation is not detected by X-ray imaging. b. The red lines show location of ureteral stent encrustation. c. The proximal coil is thick for a ureteral stent. d. The distal coil is thick for a ureteral stent.

Because three days of antibiotic treatment was ineffective, a double loop ureteral stent (Vortek® ureteral stents, 6Fr, 22 cm; Coloplast, USA) was placed in her right ureter. Following the ureteral stent placement, the pyelonephritis resolved, and she gave birth naturally at 38 weeks two days of gestation. One week after delivery, we attempted to remove the ureteral stent, because X-ray imaging and CT reveal that ureteral stone disappeared; however, the presence of encrustation on the stent complicated the procedure. X-ray imaging did not detect encrustation of the ureteral stent (Figure. 2a); however, CT showed encrustation of the proximal and distal coils, in retrospect (Figure. 2c, 2d). We explained to the patient that we do not have facilities for extracorporeal shock wave lithotrity (ESWL), which is the first choice for minimally invasive treatment of adhesions, but she strongly desired to be treated at our hospital, so we decided to perform TUL at first.

We thus performed TUL to crush and remove the ureteral stent encrustation. First, we crushed the encrustation of the proximal coil of the ureteral stent using a holmium: YAG laser (VersaPulse; Lumenis, Tel Aviv, Israel) (Figure. 2b, 3a). Second, we inserted a rigid ureteroscope into the right ureter, from the side of the ureteral stent to reach the renal pelvis, identified encrustation of the proximal coil of the ureteral stent, and crushed it (Figure. 2b, 3b). Because the lower ureteral stone could not be identified, it was assumed that it had already been excreted. The ureteral stent was successfully removed, and the crushed pieces were collected using a nitinol stone retrieval basket (Escape; Boston Scientific, Natick, MA, USA). The encrustation component was calcium phosphate, and the urinary pH during pregnancy and after delivery was 7.5.

## DISCUSSION

During pregnancy, multiple lithogenic constituents of the urine, including calcium and oxalate, increase the risk of encrustation, while multiple urinary stone inhibitors, including citrate and glycosaminoglycans, which inhibit crystal growth and aggregation and might decrease encrustation formation, also increase (3). Whether pregnancy is a risk factor for encrustation remains controversial.

In contrast, the prolonged indwelling time of ureteral stents has been reported as a risk factor for encrustation formation. El-Faqih showed that the rate of complications was as high as 76.3% when the stent was maintained for more than 12 weeks (4). Matthew R advised that the stent should be replaced at least every four months or, more ideally, every two months (5). However, the optimal schedule for ureteral stent replacement intervals is not yet established.

Urinary tract infections promote the formation of infected stones, including magnesium ammonium phosphate stones (6). Recently, it has been reported that urinary tract infections might affect the formation of non-infectious urinary stones (7). Some in vitro studies revealed that infection with non-uriealytic bacteria (primarily E. coli) promotes aggregation and growth of calcium oxalate crystals (8). Some in vivo studies of the mechanism of this effect using animal models revealed that infection by E. coli produces causes mucosal damage and an inflammatory response, releasing inflammatory proteins that promote stone formation and growth (9). On the other hand, greater pH could promote infectious and non-infectious phosphate encrustations (10). In general, grater urinary pH is associated with the formation of phosphate stones. Bauzá et al. reported that greater pH could promote infectious and non-infectious phosphate encrustations (10). They performed urinary biochemistry (24 h) and stent encrustation analysis in 90 patients requiring ureteral stent placement. As a result, patients with infectious and noninfectious phosphate encrustations had significantly higher urinary pH than patients without encrustations (10). In the present patient, the duration of the indwelling ureteral stent was not long (13 weeks), but the ureteral stent was placed for calculous pyelonephritis. Furthermore, the urinary pH during pregnancy and after delivery was 7.5. The ureteral stent encrustation was composed of calcium phosphate. Higher pH and urinary tract infections may be risk factors for phosphate encrustation formation, and further studies on the risk factors of ureteral stent encrustation are required to determine the generalizability of the findings described in this case report.

When ureteral stent removal is difficult because of encrustation, forced removal should never be performed, as this might definitively damage the ureter. Surgical treatments, including ESWL and TUL, are generally used to crush ureteral stent encrustation. In the present patient, we selected TUL to crush the encrustation of the proximal and distal coils of the ureteral stent simultaneously. Intraoperatively, the ureteroscope was inserted into the ureter without much resistance, probably because of pre-stenting.

In the present patient, radiography did not reveal ureteral stent encrustation (Figure. 2a); thus, CT may be more suitable than radiography for detecting ureteral stent encrustation. However, routine CT scans are impossible due to the risk of radiation exposure to the fetus. Although US is often used to examine pregnant women, it is not suitable for detecting ureteral stent encrustation. Further studies on the risk factors of ureteral stent encrustation and the development of tools for detecting ureteral stent encrustation that can be safely used in pregnant women are required. At present, we believe that shortening the interval between ureteral stent replacements is the most effective for preventing ureteral stent encrustation, at least in patients with factors considered to be risk factors, including grater pH and urethral stenting for obstructive pyelonephritis. However, ultimately, it is imperative to develop a ureteral stent that is free from encrustation and can be safely used in pregnant women.

## CONCLUSION

Even in pregnant patients, patients placed ureteral stents for obstructive pyelonephritis with high urine pH might need to be replaced in the short term due to concerns regarding phosphate encrustation.

## Figures and Tables

**Figure 3:**
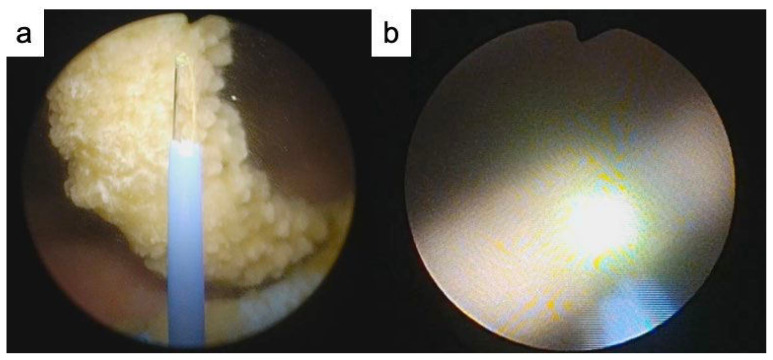
Intraoperative findings of lithotripsy a. Encrustation of the distal coil of the ureteral coil b. Encrustation of the proximal coil of the ureteral coil
